# Uromodulin modulates mitochondria and kidney tubule resilience

**DOI:** 10.1172/JCI193829

**Published:** 2025-06-16

**Authors:** Ronak Lakhia, Chunzi Song, Vishal Patel

**Affiliations:** Department of Internal Medicine and Division of Nephrology, UT Southwestern Medical Center, Dallas, Texas, USA.

## Abstract

Uromodulin is the most abundant protein in human urine, playing diverse roles, from providing frontline defense against uropathogens to regulating electrolyte balance via modulation of ion channels and cotransporters. In this issue of the *JCI*, Nanamatsu et al. unveil an alternatively spliced isoform of uromodulin that was dynamically induced in response to oxidative stress and tubular injury. Unlike the canonical secreted form, this isoform was retained in the cell, where it interacted with solute carrier proteins primarily localized to the mitochondrial membrane. Through these interactions, it modulated mitochondrial energetics and enhanced tubular cell resilience to injury. These findings broaden our understanding of uromodulin’s multifaceted functions, uncover an adaptive mechanism by which the kidney responds to cellular stress, and open avenues for therapeutic strategies targeting kidney injury and repair.

## Uromodulin in health and physiology

In 1950, immunologist Igor Tamm and nephrologist Frank Horsfall discovered a urinary protein, which they named the Tamm-Horsfall protein, that had a role as an inhibitor of viral hemagglutination (1). This protein was rediscovered in 1985 by Muchmore and Decker as an immunosuppressive, 85-kilodalton glycoprotein isolated from urine of pregnant women (2). They renamed it uromodulin, signifying its urinary origin and immunomodulatory properties. Since then, substantial advancements have deepened our understanding of this remarkable protein (3). Uromodulin is produced exclusively by kidney tubules, predominantly in the thick ascending limb (TAL) of the loop of Henle, with some contributions by the distal convoluted tubule (DCT) of the nephron. Structurally, uromodulin possesses a glycosylphosphatidylinositol (GPI) anchor signal at its C-terminus that tethers it to the apical membrane of renal epithelial cells. Proteolytic cleavage of this anchor facilitates its release into the urinary space, where uromodulin is the most abundant protein. Another key structural element is its zona pellucida (ZP) domain, located near the C-terminus, which mediates polymerization into filaments upon secretion in the urine. These filaments enable uromodulin to execute a diverse array of physiological functions. Perhaps the most well known is its role in frontline defense as a scavenger, protecting against urinary tract infections by trapping uropathogens and microbial components within the filamentous networks. The polymeric uromodulin also prevents development of kidney stones by inhibiting crystallization and plays a role in immune modulation via interaction with immune cells, maintaining urinary epithelial integrity.

Monomeric uromodulin — distinct from the polymeric form — appears to function as a signaling molecule in the TAL and DCT segments of the nephron. The TAL plays a crucial role in concentrating urine. It is impermeable to water but actively reabsorbs sodium (Na^+^), potassium (K^+^), and chloride (Cl^–^) ions from the filtrate into the surrounding interstitial fluid via the Na^+^/K^+^/2Cl^–^ (NKCC2) cotransporter. This ionic reabsorption increases the osmolarity of the medullary interstitium, generating a hyperosmotic environment that is essential for water reabsorption in downstream segments of the nephron. Located after the loop of Henle and before the collecting duct, the DCT is involved in selective reabsorption of Na^+^, calcium (Ca^2+^), and Cl^–^ ions, as well as secretion of K^+^ and hydrogen (H^+^) ions. This process is regulated by hormones such as aldosterone and parathyroid hormone, allowing fine-tuning of the filtrate and maintaining blood pressure, pH balance, and overall homeostasis. Interestingly, monomeric uromodulin directly influences ion channels (ROMK, TRPV5, and TRPM6) and cotransporters (NKCC2 and NCC), thereby modulating electrolyte transport by the TAL and DCT (4).

## Uromodulin in disease and pathology

Altered expression and structural changes in uromodulin have been implicated in a range of kidney disorders (5). The clearest example involves mutations in the *UMOD* gene, which cause autosomal dominant tubulointerstitial kidney disease (ADTKD-UMOD) — a monogenic hereditary condition characterized by early-onset hyperuricemia, gout, and progressive renal failure (6). These mutations lead to production of misfolded uromodulin protein, which accumulates within the endoplasmic reticulum, triggering cellular stress and subsequent interstitial fibrosis. Small molecules that reduce intracellular accumulation and restore the apical localization of the mutant UMOD protein have shown early promise in preclinical models (7).

Beyond inherited disorders, recent studies have identified a link between uromodulin and hypertension (8), perhaps attributable to its regulatory effects on NKCC2 and NCC cotransporters in the TAL and DCT, thereby implicating it in salt-sensitive blood pressure control. Uromodulin is also involved in the progression of chronic kidney disease (CKD) (9); decreased urinary uromodulin levels tend to correlate with reduced nephron mass and may serve as predictors of CKD progression and mortality (10). Additionally, uromodulin plays a role in acute kidney injury (AKI), where its levels fluctuate in response to tubular damage, offering promise for early diagnosis and risk stratification (11).

## Uromodulin splices its way into mitochondria during AKI

Uromodulin plays a dual role — protective under normal physiological conditions but potentially pathogenic when dysregulated — making it a potential target for diagnostic and therapeutic strategies across a wide range of renal and systemic disorders. However, beyond ADTKD-UMOD, the association between uromodulin and kidney diseases has largely been correlative, underscoring the need for direct mechanistic insights to enable development of effective, rational therapies.

In this issue of the *JCI*, Nanamatsu et al. (12) reveal a direct role for uromodulin in enhancing tubular resilience following acute injury, while also uncovering an unexpected function for this known protein. The authors began by identifying an evolutionarily conserved, alternatively spliced, in-frame variant of *UMOD* mRNA that was specifically induced in response to tubular injury across multiple mouse models and human samples. Notably, expression of this spliced *UMOD* variant correlated with injury severity and was exclusively upregulated in AKI contexts associated with recovery. In contrast, expression of full-length *UMOD* mRNA remained unchanged after injury, suggesting a distinct role for the alternatively spliced isoform in promoting tubular repair and resilience.

The first insight into the potential function of this smaller isoform arose from the observation that it lacked exon 10, which encodes the GPI anchor essential for apical membrane localization (12). Consistent with this result, Nanamatsu and coauthors found that the alternatively spliced uromodulin isoform (AS-UMOD) was retained intracellularly. However, unlike the mutant UMOD proteins linked to ADTKD, which also remain inside the cell, AS-UMOD did not trigger endoplasmic reticulum stress or cytotoxicity. Instead, AS-UMOD localized near mitochondria, where it interacted with members of mitochondrial solute carrier family 25 (SLC25) and beneficially modulated mitochondrial energetics, thereby conferring cytoprotective effects (12).

**Figure 1:**
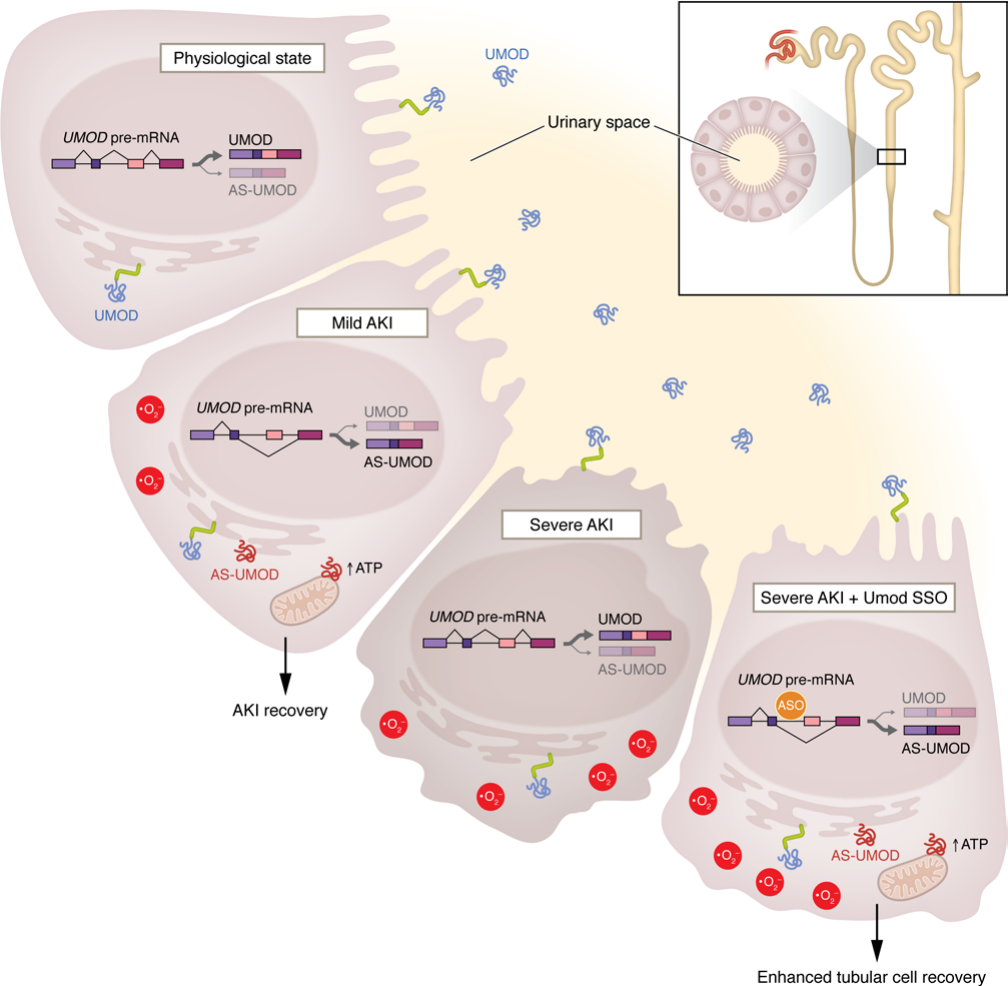
An AS-UMOD isoform of uromodulin promotes tubular recovery following injury. Under homeostatic conditions, cells in the thick ascending limb produce full-length uromodulin, which is trafficked to the apical surface or secreted into the urinary space. In response to tubular stress, such as mild AKI, TAL cells also produce AS-UMOD. This isoform is retained in the cell, where it interacts with mitochondrial transporters to enhance cellular energetics, including ATP production, and confers resilience to injury. This adaptive mechanism is lost during severe AKI. However, therapeutic administration of splice-switching antisense oligonucleotides (SSOs) can pharmacologically elevate AS-UMOD levels even in severe AKI, resulting in reduced kidney damage.

Impressively, Nanamatsu and colleagues took the next step toward testing whether this biology could be harnessed for therapeutic gain. They screened and identified a splice-switching oligonucleotide that selectively promoted AS-UMOD expression over that of the full-length uromodulin. Treatment with this oligonucleotide in a severe AKI model successfully induced AS-UMOD activation and alleviated kidney damage. Moreover, they demonstrated that the protective effect was mediated through enhanced cellular energetics in the TAL segment of the nephron (12) (Figure 1).

## Considering AS-UMOD/TAL as an axis for AKI recovery

The study by Nanamatsu et al. (12) offers several valuable insights and, as with any novel research, prompts many questions. Notably, despite its high metabolic activity and susceptibility to ischemic damage, the TAL exhibits remarkable resilience to AKI, though the underlying mechanisms remain elusive. This study suggests as one possibility that oxidative stress elicits dynamic splice-switching, leading to activation of AS-UMOD. A key unresolved process is the mechanism by which cells of the TAL sense oxidative stress and the molecular machinery needed for mediating the posttranscriptional processing of *UMOD* mRNA. Importantly, AS-UMOD was not induced during severe injury, suggesting the presence of a complex regulatory mechanism that likely depends on an oxidative stress threshold (12). A promising direction for further research would be to examine *UMOD* mRNA itself, comparing the changing repertoire of RNA-binding proteins (RBPs) and RNA chemical modifications across homeostatic, mild-stress, and severe-injury conditions.

Uromodulin is well known to modulate electrolyte transport via its action on a growing list of channels and cotransporters on the apical tubular surface. Nanamatsu and colleagues expand this functional pleiotropy by demonstrating that AS-UMOD interacts with the mitochondrial SLC25 family members. However, as is the case with apical channels, the biophysical nature of the AS-UMOD–SLC25 interaction remains unknown and is worthy of further exploration. A critical question arises as to whether enhanced activity of SLC25 family members could mimic the beneficial effects of AS-UMOD in AKI. If so, related strategies may open new therapeutic avenues for AKI management. Additionally, full-length uromodulin is thought to protect the kidney from ischemic injury, as observed in studies using *Umod*-knockout mice (13, 14), which presumably also lack AS-UMOD. Therefore, it will be important to reexamine these models to parse out the relative protective contributions of full-length uromodulin versus AS-UMOD during AKI recovery.

Much of the attention in the study of repair and regeneration following AKI has focused on the proximal tubules of the nephron. However, AS-UMOD also implicates the TAL segment in renal resilience to injury. Emerging single-cell and spatial omics technologies, currently being applied to human AKI samples, are expected to provide deeper insights into the role of the TAL in renal recovery. It is tempting to speculate that therapeutic strategies aimed at protecting the TAL may promote broader recovery across adjacent nephron segments.

Nanamatsu and authors also present compelling evidence that splice-switching oligonucleotides inducing AS-UMOD effectively reduce the severity of kidney injury in mice. This promising finding marks a crucial first step toward therapeutic development, although further optimization of oligonucleotide chemistry and delivery methods will be essential to maximize efficacy and safety.

From a broader perspective, given the unpredictability of injury onset and the rapid response required by cells, posttranscriptional regulation of preexisting RNA offers a distinct advantage over de novo gene transcription. This mechanism enables a faster cellular response to injury, suggesting that many additional regulatory axes similar to AS-UMOD remain to be discovered.

## Version 1 06/16/2025

Electronic publication
